# Prevalence of Abnormal Radiological Findings in Health Care Workers with Latent Tuberculosis Infection and Correlations with T Cell Immune Response

**DOI:** 10.1371/journal.pone.0000805

**Published:** 2007-08-29

**Authors:** Rajnish Joshi, Samir Patil, Shriprakash Kalantri, Kevin Schwartzman, Dick Menzies, Madhukar Pai

**Affiliations:** 1 Division of Epidemiology, School of Public Health, University of California at Berkeley, Berkeley, California, United States of America; 2 Department of Medicine, Mahatma Gandhi Institute of Medical Sciences, Sevagram, Maharashtra, India; 3 Parmanand D. Hinduja Hospital and Medical Research Center, Mumbai, Maharashtra, India; 4 Respiratory Epidemiology and Clinical Research Unit, Montreal Chest Institute, McGill University, Montreal, Canada; 5 Department of Epidemiology, Biostatistics and Occupational Health, McGill University, Montreal, Canada; University of California at Merced, United States of America

## Abstract

**Background:**

More than half of all health care workers (HCWs) in high TB-incidence, low and middle income countries are latently infected with tuberculosis (TB). We determined radiological lesions in a cohort of HCWs with latent TB infection (LTBI) in India, and determined their association with demographic, occupational and T-cell immune response variables.

**Methodology:**

We obtained chest radiographs of HCWs who had undergone tuberculin skin test (TST) and QuantiFERON-TB Gold In Tube (QFT), an interferon-γ release assay, in a previous cross-sectional study, and were diagnosed to have LTBI because they were positive by either TST or QFT, but had no evidence of clinical disease. Two observers independently interpreted these radiographs using a standardized data form and any discordance between them resolved by a third observer. The radiological diagnostic categories (normal, suggestive of inactive TB, and suggestive of active TB) were compared with results of TST, QFT assay, demographic, and occupational covariates.

**Results:**

A total of 330 HCWs with positive TST or QFT underwent standard chest radiography. Of these 330, 113 radiographs (34.2%) were finally classified as normal, 206 (62.4%) had lesions suggestive of inactive TB, and 11 (3.4%) had features suggestive of active TB. The mean TST indurations and interferon-γ levels in the HCWs in these three categories were not significantly different. None of the demographic or occupational covariates was associated with prevalence of inactive TB lesions on chest radiography.

**Conclusion/Significance:**

In a high TB incidence setting, nearly two-thirds of HCWs with latent TB infection had abnormal radiographic findings, and these findings had no clear correlation with T cell immune responses. Further studies are needed to verify these findings and to identify the causes and prognosis of radiologic abnormalities in health care workers.

## Introduction

More than half of all health care workers (HCWs) in the high TB-incidence, low and middle income countries (LMICs) are estimated to be latently infected with tuberculosis (TB)[1], [2] and this high prevalence is attributable to increased occupational exposure to *Mycobacterium tuberculosis,* in addition to possible exposure in the community.[1], [3] In high TB incidence countries such as India, HCWs may be repeatedly exposed to *M. tuberculosis*; previous studies have shown high prevalence of latent TB infection (LTBI), and high rates of conversions (new infections) among HCWs.[4], [5], [6] However, low-income countries often do not routinely screen HCWs for LTBI, nor implement TB infection control programs.

In high income countries, tuberculin skin test (TST) or interferon-γ release assays (IGRA) are used to screen HCWs for the presence of LTBI.[7], [8] A sizeable proportion of these HCWs are trained in high TB-incidence LMICs and have a high probability of having LTBI.[9] All individuals with positive tests, are usually evaluated for active TB disease by clinical assessment, chest radiology, sputum smears, and cultures, as indicated.[10] Radiological screening for active TB disease is recommended even in absence of any symptoms.[11] Many individuals and especially HCWs residing in LMICs have asymptomatic abnormalities on chest radiographs,[12], [13] and chest radiography is considered as a cost effective screening tool or them.[14] Previous studies have shown that the inter-observer agreement in the interpretation of TB related abnormalities on chest radiographs is variable, [15], [16] and their presence often causes alarm and results in extensive or invasive pulmonary or microbiological investigation.

We carried out this study with an aim to estimate the prevalence of radiographic abnormalities in a high TB incidence setting, and to determine the inter-observer variability in the interpretation of chest radiographs in this context. We also aimed to determine the association of asymptomatic radiological abnormalities with demographic, occupational and T-cell immune response parameters in HCWs employed in a rural, high-TB incidence setting in India.

## Materials and Methods

### Setting and study design

We evaluated chest radiographs which were obtained in a study carried out among HCWs at the Mahatma Gandhi Institute of Medical Sciences (MGIMS), Sevagram, a rural, tertiary medical school hospital in central India. Between January and June 2004, a cross-sectional study was carried out in this hospital to determine the prevalence of LTBI using TST and QuantiFERON-TB Gold In Tube (QFT), a commercially available IGRA. The design and methodology of this study have been previously described.[6] Briefly, a total of 726 HCWs with no reported history of active TB had participated in the study after signed informed consent, and 334 (46%) of these were found to have positive results by either TST (0.1 ml of 1TU PPD, reading interval 48–72 hrs,10 mm cut-off) or QFT test (ESAT 6, CFP 10, TB7.7 antigens, in-tube version, cut off value of IFN-γ≥0.35 IU/mL). As part of the study protocol (approved by the local ethics committee), these HCWs were screened for the presence of active TB by clinical examination and standard postero-anterior view chest radiographs. All symptomatic HCWs, and those who had radiological lesions suggestive of active TB disease were further investigated by sputum smears and cultures, therapeutic trial of antibiotics, and repeat chest radiographs as indicated. All asymptomatic HCWs, positive by either TST or QFT, and without any evidence of active TB were classified as having LTBI, and were offered LTBI treatment by their usual care providers. The study design was approved by the institutional review boards of MGIMS Sevagram and University of California, Berkeley.

### Interpretation of chest radiographs

All the chest radiographs from the previous study were stored, with the individual identification number (ID) as the only identifier. In India, internists are trained to read chest radiographs as part of their clinical training, and in most areas internists are the only health care providers who interpret them. In the present study two internists, each with six years of post-residency clinical experience (RJ and SP), interpreted these radiographs, independently, and blinded to any demographic or clinical data. However, these internists were aware that all radiographs were from HCWs with positive TST or QFT results. All discordant radiographs were evaluated by a third senior internist with 30 years of clinical experience (SK) whose interpretation was considered as final. We used standard criteria for reading x-rays, adapted from United States Department of State instructions for reporting of radiological abnormalities[17] to interpret the presence of each radiological abnormality. We also used International Labor Office (ILO) guidelines[18] to classify small nodular opacities according to size. Before starting the study, the use of these definitions was standardized across all observers by comparing the definitions with the images of abnormalities in a set of radiographs from the patients with active and healed TB disease, and with those in standard texts.[19] After this pilot testing, the two observers interpreted the abnormalities in the study radiographs. Based on the radiological abnormalities present, each radiograph was classified as either: 1) normal, 2) suggestive of inactive TB, or 3) suggestive of active TB (Table 1).

**Table 1:** Study definitions.

Radiological category	Definition
Normal Chest X-ray	No identifiable cardiothoracic or musculoskeletal abnormality
**Abnormalities suggestive of active tuberculosis**	
Consolidation	Opacification of airspaces within the lung parenchyma. Consolidation or infiltrate can be dense or patchy and might have irregular, ill-defined, or hazy borders.
Cavity	Lucency (darkened area) within the lung parenchyma, with or without irregular margins that might be surrounded by an area of airspace consolidation or infiltrates, or by nodular or fibrotic (reticular) densities, or both. The walls surrounding the lucent area can be thick or thin. Calcification can exist around a cavity
Tuberculoma	Round density within the lung parenchyma, also called a tuberculoma. Nodules included in this category are those with margins that are indistinct or poorly defined. The surrounding haziness can be either subtle or readily apparent and suggests coexisting airspace consolidation.
Hilar adenopathy	Enlargement of lymph nodes in one or both hila or within the mediastinum, with or without associated atelectasis or consolidation.
Pleural effusion	Presence of a significant amount of fluid within the pleural space. This finding must be distinguished from blunting of the costophrenic angle, which may or may not represent a small amount of fluid within the pleural space
Miliary	Nodules of millet size (1 to 2 millimeters) distributed throughout the parenchyma.
**Abnormalities suggestive of inactive tuberculosis**	
Fibrotic scar	Discrete linear or reticular densities within the lung. The edges of these densities should be distinct and there should be no suggestion of airspace opacification or haziness between or surrounding these densities. Calcification can be present within the lesion and then the lesion is called a “fibrocalcific” scar.
Non-calcified nodules	One or more nodular densities with distinct borders and without any surrounding airspace opacification. Nodules are generally round or have rounded edges. These features allow them to be distinguished from infiltrates or airspace opacities. To be included here, these nodules must be noncalcified.
Calcified nodules	Discrete calcified nodule or granuloma, or calcified lymph node. The calcified nodule can be within the lung, hila, or mediastinum. The borders must be sharp, distinct, and well defined. These nodules were classified according to size, based on ILO classification as less than 1.5mm, 1.5 to 3 mm, 3 to 10 mm, or larger than 10 mm
Fibrotic scar with volume loss	Discrete linear densities with reduction in the space occupied by the upper lobe. Associated signs include upward deviation of the fissure or hilum on the corresponding side with asymmetry of the volumes of the two thoracic cavities.
Nodule with volume loss	One or more nodular densities with distinct borders and no surrounding airspace opacification with reduction in the space occupied by the upper lobe. Nodules are generally round or have rounded edges.
Bronchiectasis	Bronchiectasis is bronchial dilation with bronchial wall thickening.
Pleural thickening	Irregularity or abnormal prominence of the pleural margin, including apical capping (thickening of the pleura in the apical region). Pleural thickening can be calcified.
Diaphragmatic tenting	A localized accentuation of the normal convexity of the hemidiaphragm as if “pulled upwards by a string.”
Blunt costophrenic angle	Loss of sharpness of one or both costophrenic angles. Blunting can be related to a small amount of fluid in the pleural space or to pleural thickening
**Nontubercular pulmonary abnormalities**	
**Non-pulmonary abnormalities**	

ILO = International Labor Office

### Statistical analysis

We entered the radiological data interpreted by each observer into the original study database, using ID number as the linking identifier. We used the kappa (κ) statistic to evaluate the agreement between two physicians. A kappa value of 0 indicates that the observed agreement is same as that expected by chance, and that of 1 indicates perfect agreement. The following guides were used to interpret the kappa statistic: values of <0.20 indicated poor agreement, 0.21–0.40 fair agreement, 0.41–0.60 moderate agreement, 0.61–80 good agreement, and 0.81–1.0 very good agreement.[20]

We carried out a descriptive analysis of the radiological data and estimated mean TST and QFT values for each radiological abnormality. We also determined the distribution of radiological abnormalities across TST indurations of 10 and 15 mm, and IFN-γ levels (0.35 and 0.70 IU/mL). We chose different cut-off values of these tests to determine if the radiological abnormalities were differentially distributed in individuals with a higher value. We carried out a multivariable logistic regression to determine factors associated with inactive TB (calcified nodules and other abnormalities) as determined by chest radiography. HCWs with radiological lesions suggestive of possible active TB were excluded from this analysis. We considered occupational (job category, years in service), non-occupational (age, gender, level of education), and immune response indicators (presence of BCG scar, concordance in TST and QFT results, levels of TST induration and QFT assay continuous results) as explanatory variables.

All the explanatory variables were included in the initial logistic model and a backward step wise technique was used in the selection of the final model. For a variable to be removed from the model, the *p-value* had to be >0.1.The impact of removal of each variable in the model was evaluated using the likelihood ratio test. Key explanatory variables such as age, and years in health profession were included in the final model. The fit of the final logistic model was assessed using the Hosmer-Lemeshow goodness-of-fit test.[21] The results of the final model are presented as adjusted odds ratios (OR) with 95% confidence intervals (CI). All statistical analyses used Stata (Version 9, Stata Corporation, Texas, USA).

## Results

**Figure 1:**
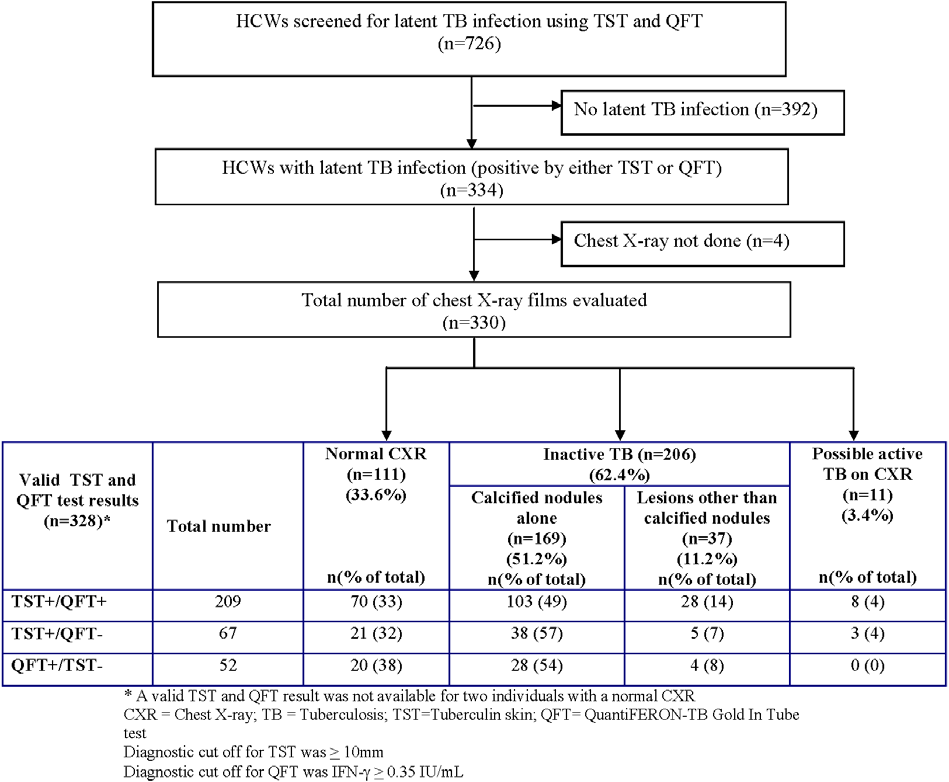
Study flow chart and distribution of individuals according to their tuberculin skin test (TST) and QuantiFERON-TB Gold assay (QFT) results.

Figure 1 shows the profile of study subjects. A total of 334 HCWs were diagnosed to have LTBI in the original study, and of these, 330 underwent chest radiography (mean age 31.5 years (standard deviation [SD] 11.9), 65% females). Of these, 328 HCWs had undergone both TST and QFT tests (209 (64%) were positive by both tests, 67 (20%) by TST alone, and 52 (16%) by QFT alone (Figure 1).

Of the 330 radiographs interpreted by two trained readers, 43 (13%) had discordant results, and the unadjusted percent agreement between the two readers was 86.9% and the agreement beyond chance was good (κ = 0.78, standard error 0.03). The agreement beyond chance was lower for the interpretation of active TB as compared to the other diagnostic categories (Table 2).

**Table 2:** Kappa statistic and percent agreement between two observers who read chest radiographs (n = 330).

Radiological diagnostic category	Agreement in interpretation of radiological findings					
	Concordant results		Discordant results		Percent agreement	Kappa (Standard Error)
	Both observers positive (n)	Both Observers negative (n)	Observer 1 positive/Observer 2 negative (n)	Observer 1 negative/Observer 2 positive (n)		
**Normal X-ray**	98	215	8	9	94.8	0.88 (0.05)
**X-ray suggestive of inactive TB**	197	111	12	10	93.3	0.85 (0.05)
**X-ray suggestive of possible active TB**	11	310	4	5	97.2	0.69 (0.05)

Overall percent agreement 86.9% (κ = 0.78, standard error 0.03)

After the discordance was resolved by the third observer, of the 330 chest radiographs 113 (34.2%) were classified as normal, 11 (3.3%) as suggestive of possible active TB, and the remaining 206 (62.4%) as inactive TB. Five individuals with lesions suggestive of inactive TB also had non-tubercular lesions (four had chronic obstructive airway disease, one had cardiomegaly) Three individuals with radiological lesions suggestive of possible active TB were symptomatic and had provided sputum specimens for microbiological evaluation, one of whom was positive for acid fast bacilli and was initiated on anti-TB treatment (prevalence of bacillary-disease being 3 per 1000). The remaining ten HCWs were closely followed, were provided with empirical, broad-spectrum antimicrobials when indicated, and resolution or non-progression of their radiological lesions was demonstrated. These individuals had no demonstrable active TB during follow-up.

All 206 HCWs with inactive TB were asymptomatic and had a total of 244 radiological findings (Table 3). Of these 206 HCWs, 169 (82%) had calcified nodules as the only abnormality. The remaining 37 HCWs (18%) had abnormalities other than calcified nodules which suggested inactive TB disease (27 of these 37 individuals also had one or more calcified nodules, so overall 196 of 206 individuals (95%) with radiological features of inactive TB had calcified nodules). Most radiographs had 2 to 5 calcified nodules between 3 and 10 mm in size. Ten of these 206 (4.8%) HCWs had fibrotic scars, all of them exceeded 2 square cm in dimension. Eight individuals had radiological evidence of costophrenic angle blunting, and underwent ultrasonography; no pleural effusion was demonstrated in any.

**Table 3:** Frequency and distribution of chest radiographic abnormalities suggestive of inactive tuberculosis (n = 206).

Abnormality*	Number of individuals with abnormality	Percent (of all abnormalities)
Calcified nodules	196	80.3
Size (of largest nodule)		
Less than 1.5mm	4	1.6
1.5 to 3 mm	24	9.8
3 to 10 mm	162	66.4
More than 10 mm	6	2.4
Number		
Single	2	0.8
2 to 5	172	70.5
More than 5	22	9.0
Fibrotic scar (all with size >2 sq. cm)	10	4.1
Non-calcified nodules‡	10	4.1
Pleural thickening	1	0.4
Diaphragmatic tenting	14	5.7
Blunt costo-phrenic angle	8	3.2
**Nontubercular pulmonary abnormality** †	4	1.6
**Non-pulmonary abnormality** §	1	0.4
Total number of abnormalities	244	

* 113 chest radiographs were interpreted as normal. 11 radiographs had lesions suggestive of possible active TB, one of whom (cavitary lesion with surrounding consolidation) was positive for acid fast bacilli. Radiological lesions in remaining ten (air space consolidation 7, hilar adeopathy 3) resolved either spontaneously or after antibiotics. A total of 244 abnormalities were reported in the remaining 206 radiographs.

‡ Of the 10 individuals with a non-calcified nodule, 4 had a single nodule, and 6 had between 2 and 5 nodules. The size of the largest nodule was between 1.5 to 3 mm in 2, and between 3 and 10 mm in 8 of them.

† The four individuals with non-tubercular pulmonary abnormality had radiological features of chronic obstructive airway disease in addition to calcified nodules suggestive of inactive tuberculosis.

§ The non-pulmonary abnormality was cardiomegaly and pulmonary hypertension in addition to calcified nodules suggestive of inactive tuberculosis.

Contrary to our expectations calcified nodules were seen in 36 of 60 (60%) of HCWs who were between 18 to 20 years of age. Five of them (8.3%) also had non-calcified lesions suggestive of inactive TB, and the only HCW with microbiologically confirmed active TB was also a medical student in this age group.

**Figure 2:**
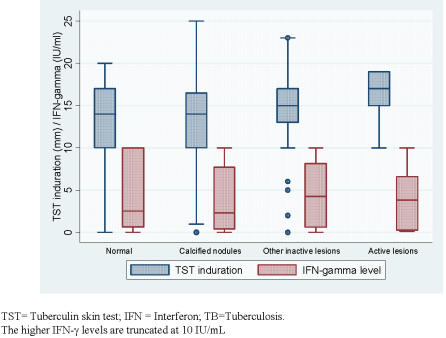
Distribution of tuberculin skin test indurations, and interferon gamma levels across different radiological categories. With the individuals with a normal CXR as the reference, the mean TST indurations (mm) in the groups with calcified nodules alone, and other inactive lesions were not statistically significant (p values 0.98, and 0.34). TST induration was significantly higher in HCWs with radiological lesions suggestive of possible active TB (p=0.03), as compared to those with normal CXR. With the individuals with a normal CXR as the reference, the mean IFN-g values (IU/mL) in the groups with calcified nodules alone, other inactive lesions, and lesions suggestion of active TB were not statistically significantly (p values 0.34, 0.63 and 0.91 respectively)

The mean TST indurations and IFN-γ levels in the individuals with radiological features of calcified nodules, other non-calcified abnormalities suggestive of inactive TB, were not statistically different from those with normal chest radiographs (Figure 2). Mean TST indurations and IFN-γ values in HCWs with calcified nodules were similar to those with normal chest radiographs (p = 0.98, and 0.34 respectively). As compared to HCWs with normal chest radiographs, those with non-calcific nodules, and fibrotic scar, had higher but statistically non-significant TST indurations (p = 0.06, 0.07 respectively). The IFN-γ values were however similar across these groups (p value between 0.31 and 0.90) (Table 4)

**Table 4:** Distribution of tuberculin skin test indurations, and interferon gamma levels across different radiological subgroups

Abnormality	Total	TST indurations (in mm)			IFN-γ values (in IU/mL)		
		Median (IQR)	Above 10 n (% of total)	Above 15 n (% of total)	Median (IQR)	Above 0.35 n (% of total)	Above 0.70 n (% of total)
Normal CXR	111*	14 (10–17)	91 (81.9)	52 (46.8)	2.5 (0.6–10)	92 (82.8)	82 (73.8)
Calcified nodules alone	169	14 (10–17)	140 (82.8)	73 (43.1)	2.3 (0.4–7.7)	132 (78.1)	111 (65.6)
Lesions other than calcified nodules suggesting inactive TB	37	15 (13–17)	33 (89.1)	20 (54.0)	4.2 (0.6–8.1)	32 (86.4)	27 (72.9)
Blunt costo-phrenic angle	7	13 (6–15)	5 (71.4)	2 (25)	6.2 (3.9–10)	6 (85.7)	6 (85.5)
Diaphragmatic tenting (DT) alone	10	13 (10–16)	8 (85.7)	5 (50)	3.2 (0.3–7.8)	8(80)	6 (60)
DT with blunt costo-phrenic angle	1	14	1 (100)	0 (0)	1.5	1 (100)	1 (100)
DT with non-calcified nodule	3	17 (14–17)	3 (100)	2 (66.7)	4.2 (0.6–6.6)	3 (100)	3 (100)
Non-calcified nodules alone	5	16 (14–17)	5 (100)	3 (60)	2.6 (0.6–5.1)	5 (100)	3 (60)
Fibrotic scar (all with size >2cm)	10	15 (14–17)	10 (100)	7 (70)	3.8 (2.2–8.1)	8 (80)	8 (80)
Pleural thickening	1	17	1 (100)	1 (100)	10	1 (100)	1 (100)
Lesions suggesting active TB	11	17 (15–19)	11 (100)	9 (81.8)	3.8 (0.2–6.6)	8 (72.7)	8 (72.7)
Consolidation alone	7	16 (14–19)	7 (100)	5 (71.4)	3.3 (0.1–6.6)	4 (57.1)	4 (57.1)
Hilar adenopathy	3	17 (16–19)	3 (100)	3 (100)	5.9 (1.1–10)	3 (100)	3 (100)
Cavity and consolidation	1†	18	1 (100)	1 (100)	4.7	1 (100)	1 (100)

* A valid TST and QFT result was not available for two individuals with a normal CXR

† This individual had microbiologically confirmed pulmonary tuberculosis The higher IFN-γ levels are truncated at 10 IU/mL

We determined risk factors associated with inactive TB (calcified nodules, and other abnormalities) as determined by radiology and performed a multivariable logistic regression, with different occupational, non-occupational and immune response indicators as the covariates. Although higher number of years in health profession were associated with higher odds of abnormalities other than calcified nodules, none of these covariates were statistically significant (Table 5).

**Table 5:** Covariates associated with inactive tuberculosis vs. normal chest radiographs*

Covariates	Total (n = 330)	Inactive TB (n = 206)					
		Calcified nodules alone (n = 169)			Lesions other than calcified nodules (n = 37)		
		n (% of total)	Unadjusted OR (95% CI)	Adjusted OR (95% CI)	n (% of total)	Unadjusted OR (95% CI)	Adjusted OR (95% CI)
***Age***							
18–20	60	36 (60)	1	1	3 (5)	1	1
21–30	135	66 (48.8)	0.7 (0.35–1.28)	0.6 (0.22–1.41)	9 (6.7)	1.1 (0.27–4.47)	0.5 (0.06–3.35)
31–40	53	29 (54.7)	1.3 (0.55–3.03)	1.0 (0.22–4.46)	9 (16.9)	4.8 (1.1–21.25)	0.1 (0.004–3.76)
41 or more	82	38 (46.3)	1.0 (0.47–2.13)	0.8 (0.16–4.00)	16 (19.5)	5.1 (1.29–20.04)	0.1 (0.003–3.15)
***Gender***							
Female	213	108 (50.7)	1	1	20 (9.4)	1	1
Male	117	61 (52.1)	1.2 (0.72–2.0)	1.3 (0.66–2.47)	17 (14.5)	2.0 (0.95–4.31)	2.7 (0.76–9.92)
***Education level***							
Medical, master's or bachelor's degree	244	128 (52.4)	1		22 (9.0)	1	
High school or lower	86	41 (47.6)	1.1 (0.61–1.87)		15 (17.4)	2.5 (1.16–5.58)	
***Job category***							
Medical students	60	35 (58.3)	1	1	3 (5)	1	1
Nursing students	49	22 (44.8)	0.5 (0.22–1.11)	0.6 (0.25–1.51)	2 (4.1)	0.5(0.08–3.50)	0.9 (0.80–7.18)
Interns	12	7 (58.3)	0.8 (0.22–2.85)	1.1 (0.23–4.99)	0 (0)		
Residents	9	5 (55.5)	0.7 (0.17–2.96)	2.0 (0.43–9.32)	0 (0)		
Nurses	96	50 (52.0)	1.1 (0.53–2.27)	2.2 (0.72–6.58)	14 (14.6)	3.3 (0.80–13.3)	3.3 (0.37–28.50)
Laboratory staff	28	13 (46.4)	0.8 (0.30–2.27)	1.3 (0.33–5.07)	6 (21.4)	4.4 (0.90–21.87)	2.2 (0.23–19.97)
Orderlies	69	34 (49.2)	0.9 (0.44–2.11)	1.4 (0.45–4.39)	12 (17.4)	4.3 (1.06–17.57)	1.6 (0.20–13.63)
Faculty	7	3 (42.8)	0.4 (0.08–2.11)	0.6 (0.25–1.51)	0 (0)		
***Years in service***							
<1 year	25	15 (60)	1	1	1 (4)	1	1
2–5 years	103	59 (57.2)	0.9 (0.38–2.40)	1.1 (0.36–2.76)	5 (4.8)	1.2 (0.12–11.73)	1.5 (0.12–18.25)
6–10 years	74	33 (44.5)	0.6 (0.22–1.51)	0.5 (0.18–2.15)	6 (8.1)	1.3 (0.13–12.80)	1.8 (0.07–40.25)
11 years or more	128	62 (48.4)	1.1 (0.44–2.85)	0.6 (0.19–4.00)	25 (19.5)	7.1 (0.84–59.60)	23.3 (0.43–1233)
***BCG scar***							
Absent	99	57 (57.5)	1		8 (8)	1	
Present	231	112 (48.4)	0.74 (0.44–1.25)		29 (12.5)	1.4 (0.56–3.32)	
***TST and QFT results***							
Discordant	118	65 (55.0)	1		9 (7.6)	1	
Concordant	209	103 (49.2)	0.9 (0.56–1.52)		28 (13.4)	1.8 (0.78–4.23)	
***TST induration (mm)***							
Less than 10	52	28	1		4	1	
10 to 14.9	121	67	1.22 (0.61–2.46)		13	1.66 (0.48–5.77)	
15 to 19.9	137	65	1.00 (0.50–2.00)		17	1.84 (0.55–6.19)	
20 or more	20	9	0.80 (0.26–2.44)		3	1.87 (0.34–10.33)	
***QFT result (IU/mL)***							
Less than 0.35	66	37	1		5	1	
0.35 to 1.99	81	43	0.78 (0.38–1.59)		6	0.81 (0.21–3.01)	
2.00 to 3.99	41	21	0.99 (0.40–2.41)		6	2.10 (0.52–8.36)	
4.00 to 5.99	36	17	0.96 (0.37–2.48)		7	2.94 (0.74–11.60)	
6.00 to 7.99	18	10	1.41 (0.39–5.08)		3	3.15 (0.52–18.80)	
8.00 or more	88	41	0.66 (0.33–1.33)		10	1.20 (0.36–3.99)	

* Individuals who had radiological features of active tuberculosis (n = 11) were excluded from this analysis. A total of 206/319 remaining individuals had radiological features of inactive tuberculosis. Of these 169 had calcified nodules as the lone abnormality and 37 had non-calcified lesions (27 individuals in this group also had calcified nodules)

## Discussion

### Principal findings

In a rural hospital setting in India, only one-third of all health-care workers with a positive test for LTBI had a normal chest radiograph, and a majority of the remaining HCWs had chest radiographs consistent with inactive TB. None of the HCWs had reported a history of prior active TB. A majority of inactive lesions were in the form of small, multiple calcified lesions. In the absence of a comparison group of non-HCWs, it is unclear if the high prevalence of abnormalities was due to occupational TB exposure. The prevalence of radiologically-active TB disease in our study was 33 per 1000, which is much higher than the previous estimates from community based mass-miniature radiography surveys done in 1955–57 in India (13.5 to 26.6 per 1000 population).[22]. Since mass radiography surveys have been discontinued, there are no recent community based data on prevalence of radiological lesions in individuals with inactive TB in India for comparison. The prevalence of smear-positive TB disease in our study (3 per 1000) was however similar to the previously available community estimates (2.4 to 8.3 per 1000).[22] However as compared to previous studies done in immigrants from different LMIC [13], [23], [24], [25], [26], [27], [28], [29], [30], the prevalence of radiological inactive TB in our study population was much higher.

There was a good inter-observer agreement between the two physicians who interpreted the radiographs. This is not consistent with previous studies where the inter-observer agreement in interpreting the chest radiographs has been poor.[15], [16] We used standardized definitions, pilot tested the radiological criteria, and this may have resulted in a high degree of inter-observer agreement. It has been shown that such interpretation protocols improve the reading of chest radiographs, which form an important part of TB screening programs.[13], [31]

### Possible explanations for radiologic abnormalities

It is well known that chest radiography for TB lacks specificity. Radiologic lesions suggestive of TB are also noticed in conditions such as histoplasmosis, tropical eosinophilia, pneumoconiosis, siderosis, sarcoidosis, hypersensitivity pneumonitis and vasculitis. The community prevalence of these conditions among Indian adults is not known. Since in our study, all the subjects were HCWs with positive tests for LTBI and had no other reported occupational exposures, the radiologic abnormalities are probably a reflection of their recent or past exposures to *M. tuberculosis*. It is, however, not possible to rule out community exposures that may cause radiological lung abnormalities, especially among those with calcified lesions. In a previous cohort study at our hospital, the annual risk of new TB infection (ARTI) was estimated to be about 5% in a cohort of young medical and nursing trainees, and this suggests a high rate of TB exposure in this population.[4] It is plausible that repeated exposures may result in primary lung lesions that are often contained without the development of clinical disease.

Alternatively, it is plausible that at least some of the HCWs had primary TB (primary complex) since childhood. Some HCWs may have had known active TB in the past, but did not report this because of stigma. Of the 206 HCWs with radiologically inactive TB, 80 (38.8%) were in the healthcare profession for 5 years or less. None of the variables indicating a higher occupational exposure (such as duration of service as a HCW, or nature of job) were significantly associated with the presence of inactive TB lesions on multivariate analysis. The lack of statistical association and short duration of service for at least one-third HCWs, could mean that these individuals may have acquired the lesions in childhood, and their primary complex lesions had healed with a residual calcification.

Initial infection with *M. tuberculosis* results in deposition of a small number of bacilli in the intra-alveolar space, where they attract a non-specific inflammatory infiltrate, presenting radiologically as a small sublobar or subpleural focus of consolidation.[32] In two-thirds of exposed individuals this focus resolves without radiological sequelae, and in about one-third a radiologically visible scar persists which may progress to primary TB, or may self heal and get calcified.[33] Nodular subpleural (Ghon's focus) or parenchymal calcifications (Simon's focus), non-calcified nodules (tuberculomas) and fibrotic scars are all considered as radiological features of inactive or healed primary TB.[32], [33], [34] Viable intracellular mycobacteria may persist in some of these sites, and may subsequently re-activate causing post primary (reactivation) TB.[35] This widely believed concept that primary TB represents early disease, and reactivation is a late feature has recently been challenged by studies which used molecular epidemiology to determine the duration of acquisition of TB infection.[36] This study suggests that the radiological features in primary and reactivation TB represent differential host response to infection, rather than a temporal sequence of events. It is unclear if this also holds true for different radiological subtypes of LTBI.

In a person with a newly acquired LTBI, the lifetime risk to develop reactivation is about 5–10 percent.[37] Such risk estimates vary depending on the immune status of the individual, and gradually decline as the time elapsed since infection increases.[38] It is known that the individuals with radiological lesions suggesting inactive TB have a higher risk of developing TB disease as compared to those with normal chest radiographs. [39] Of the radiological subtypes of inactive TB disease, presence of a fibrotic lesions of more than 2 square cm in size, is a known risk factor for reactivation.[40] Calcification is generally considered as a hallmark of well contained infection but is not a guarantee of clinical quiescence.[41] It remains to be determined whether, in addition to radiological subtypes, IFN-γ levels are important in risk stratification of individuals with LTBI. Although IGRAs are promising tests for LTBI, it is unclear how absolute IFN-γ levels can be used as potential markers of active TB and for predicting the progression from LTBI to TB disease.[42]

### Correlation between radiologic findings and interferon-γ responses

Previous studies have attempted to correlate T cell IFN-γ levels with the extent of radiological disease in patients with active TB.[43], [44], [45] and two of these studies did not find any significant correlation. To our knowledge, no study has explored the correlation of IFN-γ levels with radiological lesions in individuals with latent TB. In the present study, the average IFN-γ level as well as the size of TST indurations were similar in individuals with normal and abnormal chest radiographs. This could be either due to poor sensitivity of chest radiographs in detecting small active lesions, or lack of any true relation between immunological response to specific TB antigens and nature or extent of lung lesions. It has been previously shown that the size of TST induration, beyond a certain threshold, has no clear correlation with the extent of radiological disease, [46] and our study suggests that this may hold true for IFN-γ as well. However, since our study was cross-sectional, it is impossible to capture changes in the correlation between radiologic findings and T-cell immune responses. Serial testing studies are needed to better understand changes in T-cell responses over time and to determine the predictive value of changes in T-cell responses. [8]

Calcified nodules are produced in an attempt to heal the lesions caused by TB infection [34] and in this study we explored if abnormalities other than calcified nodules (such as fibrotic scars, or non-calcific nodules etc) represent a different immunological subtype. The number of HCWs with such lesions in this study was too small to determine any significant difference. However our analysis does suggest that in terms of TST indurations, the radiological pattern of calcified nodules is closer to normal chest radiographs, and fibrotic scars or non-calcified nodules being closer to abnormalities suggestive of active TB. No such pattern was however detectable with more specific QFT test results.

### Study limitations

Our study had several limitations. Firstly, all study participants were HCWs positive by TST or QFT, and thus we had no comparison group of HCWs without LTBI, or non-HCWs (ie. adults working in occupations other than healthcare). The absence of a comparison group makes if difficult to determine if the high prevalence of radiographic abnormalities was due to community or occupational exposure. The two physicians who interpreted radiographs could have over-reported the findings as they were aware that all HCWs had LTBI. This bias could have potentially been minimized if these radiographs were mixed with those from non-HCWs. Secondly, we did not include a cohort of adults who were working in occupations other than health-care. The inclusion of such a group would have enabled us to compute the excess prevalence of radiologic abnormalities beyond the background community prevalence. Some end-on vessels could have been over-reported as calcified nodules, since it is difficult to distinguish between the two on standard PA view chest radiographs.

The number of HCWs with radiological lesions other than calcified nodules was small, and this could have led to a lack of precision in measurement of effect in the univariate and multivariable models. Lastly, all radiographs were read by internists, not radiologists or pulmonologists. Chest-radiographs in rural India are usually interpreted by internists and family physicians, and therefore our study is reflective of the Indian setting. Despite these limitations, our study provides useful data on prevalence of radiologic abnormalities among HCWs working in a high TB incidence setting. Our data also provide, for the first time, information on lack of correlation between radiologic findings and T cell immune responses in individuals with LTBI. Our results, however, must be considered preliminary and have to be confirmed in other studies.

### Conclusions

Our findings suggest that the majority of asymptomatic HCWs with LTBI in a high TB-incidence country have abnormal chest radiographs suggestive of inactive TB. Such a high prevalence of abnormal radiographic findings may reflect a combination of previous and recent exposures to *M. tuberculosis*. Presence of these features is not explained by demographic, occupational or immune response correlates. Further research is needed to identify the specific causes and prognosis of radiologic lesions among HCWs in high TB incidence settings, and to study the correlations between radiologic lesions and cellular immune responses. If future studies show a high prevalence of inactive TB among HCWs in developing countries, there may be a strong case for period radiographic screening of HCWs.
